# Urgent endoscopic retrograde cholangiopancreatography improves clinical outcomes in acute cholangitis from choledocholithiasis: a propensity score-matched study

**DOI:** 10.1007/s00464-025-12233-y

**Published:** 2025-09-29

**Authors:** Ronnakorn Kongsakon, Manus Rugivarodom, Pochamana Phisalprapa, Khemajira Karaketklang, Phunchai Charatcharoenwitthaya, Nonthalee Pausawasdi

**Affiliations:** 1 Siriraj GI Endoscopy Center, Faculty of Medicine Siriraj Hospital, Mahidol University, Bangkok, Thailand; 2 Division of Gastroenterology, Department of Medicine, Faculty of Medicine Siriraj Hospital, Mahidol University, Bangkok, Thailand; 3 Division of Ambulatory Medicine, Department of Medicine, Faculty of Medicine Siriraj Hospital, Mahidol University, Bangkok, Thailand; 4 Department of Medicine, Faculty of Medicine Siriraj Hospital, Mahidol University, Bangkok, Thailand

**Keywords:** Urgent ERCP, Acute cholangitis, CBD stones, Choledocholithiasis

## Abstract

**Background:**

Early endoscopic retrograde cholangiopancreatography (ERCP) with biliary drainage is recommended for acute cholangitis based on disease severity. However, the optimal timing of ERCP remains unclear. This study aimed to evaluate the impact of urgent ERCP (≤ 24 h) on clinical outcomes in patients with common bile duct (CBD) stone-related acute cholangitis.

**Methods:**

A retrospective cohort study was conducted among patients who underwent ERCP for acute cholangitis due to CBD stones between 2008 and 2017. Patients were categorized according to ERCP timing: urgent (≤ 24 h) and non-urgent (> 24 h). Outcomes included in-hospital mortality, organ failure at 72 h, length of hospital stay, procedure-related complications, and 30-day readmission. Propensity score matching (PSM) was applied to balance baseline characteristics, including age, sex, comorbidities, Charlson comorbidity index, and cholangitis severity according to the Tokyo Guidelines 2018.

**Results:**

Among 455 eligible patients, 191 matched pairs were analyzed. The mean age was 66 ± 16 years, and 50% were male. Among them, 21.5% had severe cholangitis, 40.4% moderate, and 38.1% mild disease. Following matching, patient characteristics of the two groups were balanced, except for a higher percentage of patients with moderate cholangitis in the non-urgent group. In-hospital mortality was significantly lower in the urgent ERCP group (0.5% vs 21%; adjusted OR 0.09; 95% CI: 0.01–0.73; *p* = 0.024). Median hospital stay was shorter (5 vs 8 days; *p* < 0.001), while stone clearance rates (approximately 75%), persistent organ failure, procedural complications, and readmission were comparable between groups. Subgroup analysis revealed a mortality benefit of urgent ERCP in moderate (*p* < 0.01) and severe (*p* = 0.024) cholangitis, but not in mild cases.

**Conclusions:**

Urgent ERCP within 24 h significantly reduces in-hospital mortality and shortens hospitalization in patients with moderate to severe cholangitis due to CBD stones. These findings support early intervention as a key component of management in this population.

**Graphical Abstract:**

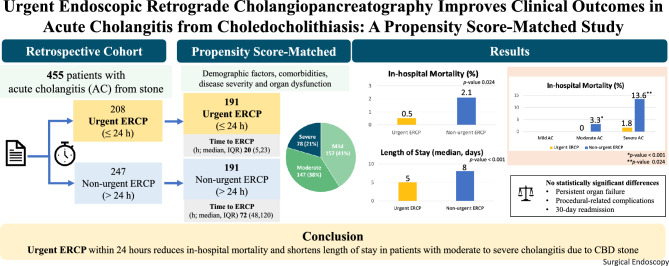

**Supplementary Information:**

The online version contains supplementary material available at 10.1007/s00464-025-12233-y.

Acute cholangitis is a potentially life-threatening condition caused by bacterial infection in the setting of biliary obstruction, most commonly due to choledocholithiasis. Without prompt treatment, the condition can progress rapidly to sepsis and multi-organ failure, leading to high mortality rate [1]. Standard management includes intravenous fluid resuscitation, broad-spectrum antibiotics, and biliary drainage [2, 3]. Endoscopic retrograde cholangiopancreatography (ERCP) remains the preferred modality for biliary decompression, offering both diagnostic and therapeutic benefits [4].

While ERCP is widely accepted as the cornerstone of cholangitis management, the optimal timing of the procedure remains a subject of ongoing debate. Experts generally recommend early biliary drainage based on disease severity; however, they do not clearly define what “early” or “urgent” intervention means. Several retrospective studies have suggested that delayed ERCP, particularly beyond 48–72 h, are associated with prolonged hospitalization and high rates of poor clinical outcomes, including in-hospital mortality, persistent organ failure, and intensive care unit stay [5]. Some data indicate that ERCP within 24 h of admission may offer additional survival benefits [6], particularly in moderate to severe cases, yet definitive evidence remains limited.

Given the lack of consensus on the ideal timing of ERCP and the potential clinical implications, this study aimed to evaluate the clinical outcomes of patients with acute cholangitis due to choledocholithiasis who underwent ERCP within 24 h after admission compared to those who received the procedure later. We selected a 24-h threshold for 'urgent' ERCP because it aligns with emerging evidence showing potential benefits, is operationally feasible within most hospital workflows, and reflects a practical window for early biliary decompression. We hypothesized that urgent ERCP would be associated with lower in-hospital mortality and improved clinical outcomes, particularly in patients with more severe disease.

## Material and methods

### Study design

This retrospective cohort study was conducted using the ERCP database from a tertiary care center. It was approved by the Institutional Review Board number 430/2562 (EC3); Certificate of Approval Si 662/2019. The study was designed and reported in accordance with strengthening the reporting of cohort, cross-sectional, and case–control studies in surgery (STROCSS) 2021 guidelines (Supplementary Data 1).

### Study population

Patient data were extracted from the electronic-based admission records of individuals admitted and subjected to ERCP between January 2008 and December 2017. The database contained records of 4,101 ERCP procedures performed during the study period. The study included patients diagnosed with acute cholangitis caused by choledocholithiasis who met the TG13/18 criteria [7]. Exclusion criteria encompassed patients with concomitant diagnoses of acute cholecystitis, liver abscess, pancreatitis, Mirizzi syndrome, or gastrointestinal obstruction.

A total of 4101 patients who underwent ERCP procedures during the study period, 784 patients were diagnosed with acute cholangitis. Among them, 191 had cholangitis due to causes other than choledocholithiasis, and 138 was excluded due to concurrent condition mentioned in the exclusion criteria. Thus, the study enrolled a total of 455 patients, who were subsequently categorized into two groups: urgent (ERCP performed within 24 h of admission) and non-urgent (ERCP performed more than 24 h after admission).

Clinical and laboratory data collected during presentation and admission were obtained from electronic medical records. To assess the severity of the disease, vital signs, including heart rate, blood pressure, and respiratory rate, as well as the presence of organ failures, were evaluated. These parameters align with the Tokyo Guideline established in 2018. Data regarding the time of hospital admission and ERCP were extracted from medical records to determine the timing of ERCP. Information on procedures performed during ERCP were retrieved from the ERCP database.

### Management of acute cholangitis caused by choledocholithiasis

A standardized approach, based on the Tokyo Guideline 2018, was employed for the initial resuscitation and risk stratification of all patients. To ensure optimal care, intravenous fluid and antibiotic therapy were administered based on individual patient needs before undergoing ERCP. ERCP procedures were performed by experienced interventional endoscopists using standard endoscopic equipment.

The biliary cannulation was performed using the wire-guided technique with efforts made to minimize the use of contrast. The choice between stent placement or endoscopic biliary sphincterotomy followed by stone extraction was at the discretion of the endoscopist, considering the specific circumstances of each case.

### Definition of outcomes

The primary outcome was in-hospital mortality. Secondary outcomes included persistent organ failure at 72 h after admission, the length of stay, complete stone removal rate, intervention-related complications, and readmission rates. Persistent organ failure was defined as the occurrence of any of the following organ system dysfunction at 72 h, based on the severity grading of acute cholangitis from TG18; cardiovascular dysfunction (hypotension requiring dopamine > 5 µg/kg/min or any dose of norepinephrine), neurological dysfunction (disturbance of consciousness), respiratory dysfunction (PaO2/FiO2 ratio < 300), renal dysfunction (oliguria, serum creatinine > 2.0 mg/dl), hepatic dysfunction (INR > 1.5), and hematological dysfunction (platelet count < 100,000/mm^3^). Intervention-related complications included post-ERCP pancreatitis, post-ERCP cholangitis, clinically significant bleeding, and perforation.

### Statistical analysis

Patient demographics and clinical characteristics were summarized using descriptive statistics. Continuous variables were expressed as mean ± standard deviation for normally distributed data or median with interquartile range for non-normally distribution data. Group comparisons for continuous variables were performed using the independent t-test or Mann–Whitney U-test, as appropriate. Categorical data were reported as frequencies and percentages, and differences between groups were assessed using the chi-square test or Fisher's exact test, as appropriate.

To minimize confounding due to non-random treatment allocation, propensity score matching (PSM) was applied. Propensity scores were derived from a multivariable logistic regression model that included demographic factors, comorbid conditions, Charlson comorbidity index, disease severity, and organ dysfunction based on the Tokyo Guidelines 2018. A 1:1 nearest-neighbor matching without replacement was performed. Subgroup analysis was conducted based on TG13/18 severity grading (mild, moderate, severe). All statistical tests were two-sided, and a *p* value < 0.05 was considered statistically significant. Analyses were carried out using IBM SPSS Statistics, version 20 (IBM Corp., Armonk, NY, USA).

## Results

### Baseline characteristics

Of the 455 patients admitted with acute cholangitis, 208 (46%) patients underwent ERCP within 24 h of admission, while 247 (54%) patients received the procedure after 24 h. Overall, 98 patients (21.5%) presented with severe cholangitis, 184 (40.4%) with moderate, and 173 (38.1%) with mild disease. The mean age was 66 ± 16 years, and approximately half of the cohort were male. The Charlson comorbidity index was slightly higher in the urgent ERCP group (4.2 ± 2.6) compared to the delayed group (3.7 ± 2.8), though the difference was not statistically significant. Renal dysfunction at presentation was more common in the urgent ERCP group (13.9% vs 6.1%, *p* = 0.005), while other clinical parameters showed no significant variation. The prevalence of most comorbidities was similar between groups, with the exception of dyslipidemia (34.8% vs 25.5%, *p* = 0.031) and malignancy (9.7% vs 4.8%, *p* = 0.047), both of which were more frequent in the non-urgent ERCP group. The proportions of patients with severe cholangitis (26.0% vs 17.8%, *p* < 0.035) and mild cholangitis (44.2% vs 32.8%, *p* = 0.012) were higher in the urgent group, whereas a higher percentage of patients with moderate cholangitis was observed in the non-urgent group (49.4% vs 29.8%, *p* < 0.001). The median time from admission to ERCP was 19.5 h, with an interquartile range of 5–23 h, in the urgent group and 74 h, with an interquartile range of 48–120 h, in the non-urgent group. The majority of patients received cephalosporin-based antibiotics (75.3% in the urgent group vs 70.2% in the non-urgent group), followed by carbapenems (17.8% vs 22.6%) and piperacillin/tazobactam (4.0% vs 5.3%), with no significant difference between groups. Other baseline characteristics were comparable, as detailed in Table 1. In non-severe cases (mild and moderate cholangitis), cephalosporins were used most frequently (75.1%), whereas carbapenem and piperacillin/tazobactam were used in 17.9% and 4.2%, respectively. In contrast, among severe cases, cephalosporin use was lower (65.3%), while the use of carbapenem (27.6%) and piperacillin/tazobactam (6.1%) was higher. Notably, the difference in carbapenem use between non-severe and severe cases reached statistical significance (*p* = 0.035).

**Table 1:** Baseline characteristics of the patients before propensity score matching

Characteristics	Urgent ERCP	ERCP > 24 h	*p* value
	(*n* = 208)	(*n* = 247)	
Male gender, *n* (%)	98 (47.1)	130 (52.6)	0.259
Age, years	64.2 ± 17.8	66.4 ± 16.0	0.155
Body weight (kg)	61.9 ± 11.1	62.8 ± 11.3	0.419
Height (cm)	159.6 ± 6.9	159.7 ± 6.8	0.919
Body mass index, kg/m^2^	24.3 ± 4.1	24.6 ± 3.9	0.49
Comorbidities, n (%)			
Diabetes	49 (23.6)	75 (30.4)	0.104
Hypertension	102 (49.0)	142 (57.5)	0.072
Dyslipidemia	53 (25.5)	86 (34.8)	0.031
Cirrhosis	7 (3.4)	11 (4.5)	0.553
Cerebrovascular disease	7 (3.4)	14 (5.7)	0.244
Cardiovascular disease	17 (8.2)	14 (5.7)	0.165
Chronic kidney disease	20 (9.6)	20 (8.1)	0.569
Chronic pulmonary disease	7 (3.4)	7 (2.8)	0.744
Malignancy	10 (4.8)	24 (9.7)	0.047
Charlson comorbidity index	4.2 ± 2.6	3.7 ± 2.8	0.106
Clinical parameters at presentation, n (%)			
Total bilirubin > 5 mg/dl	78 (37.5)	107 (43.3)	0.208
Cardiovascular dysfunction	28 (13.5)	23 (9.3)	0.162
Neurological dysfunction	16 (7.7)	11 (4.5)	0.145
Respiratory dysfunction	22 (10.6)	19 (7.7)	0.284
Renal dysfunction	29 (13.9)	15 (6.1)	0.005
Hepatic dysfunction	12 (5.8)	14 (5.7)	0.963
Hematologic dysfunction	13 (6.3)	11 (4.5)	0.393
Cholangitis severity, n (%)			
Severe	54 (26.0%)	44 (17.8%)	0.035
Moderate	62 (29.8%)	122 (49.4%)	< 0.001
Mild	92 (44.2%)	81 (32.8%)	0.012
Time to ERCP (h), median (IQR)	19.5 (5, 23)	74 (48, 120)	
Antibiotics, n (%)			
Cephalosporin based	186 (75.3%)	146 (70.2%)	0.221
Piperacillin/tazobactam	10 (4.0%)	11 (5.3%)	0.530
Carbapenem	44 (17.8%)	47 (22.6%)	0.204
Others	7 (2.8%)	4 (1.9%)	0.529

*h* hours, *IQR* interquartile rage

Data are presented as number of patients (%) unless otherwise stated. Mean values are presented as mean ± SD. Median values are presented as median (interquartile range)

After propensity score matching, each cohort consisted of 191 patients. Baseline demographics were balanced between the urgent ERCP group and the group undergoing ERCP more than 24 h after presentation (Table 2). There were no significant differences in age, sex, BMI, or comorbidity profiles, including rates of diabetes, hypertension, dyslipidemia, cirrhosis, cardiovascular and cerebrovascular disease, chronic kidney or pulmonary disease, and malignancy. The Charlson comorbidity index and initial clinical parameters, such as total bilirubin > 5 mg/dL and organ dysfunction across cardiovascular, neurological, respiratory, renal, hepatic, and hematologic systems, were similarly distributed between groups. Severity classifications revealed a higher proportion of moderate cases in the non-urgent ERCP group (45% vs 31.9%, *p* = 0.009), while the proportions of severe and mild cholangitis were comparable between the two groups. Antibiotic regimens were also similar, with no significant differences in the distribution of cephalosporin-based, carbapenem, or piperacillin/tazobactam use between the groups.

**Table 2:** Baseline characteristics of the matched cohorts after propensity score matching

Characteristics	Urgent ERCP	ERCP > 24 h	*p* value
	(*n* = 191)	(*n* = 191)	
Male gender, *n* (%)	92 (48.2)	96 (50.3)	0.682
Age, years	64.2 ± 18.0	65.1 ± 16.5	0.627
Body weight (kg)	62.2 ± 11.4	62.7 ± 10.5	0.650
Height (cm)	159.7 ± 7.1	159.3 ± 6.6	0.564
Body mass index, kg/m^2^	24.4 ± 4.2	24.7 ± 3.8	0.470
Comorbidities, *n* (%)			
Diabetes	48 (25.1)	52 (27.2)	0.642
Hypertension	95 (48.0)	103 (52.0)	0.413
Dyslipidemia	50 (26.2)	53 (27.7)	0.729
Cirrhosis	7 (3.7)	7 (3.7)	1.000
Cerebrovascular disease	7 (3.7)	7 (3.7)	1.000
Cardiovascular disease	15 (7.9)	15 (7.9)	1.000
Chronic kidney disease	18 (9.4)	16 (8.4)	0.719
Chronic pulmonary disease	7 (3.7)	6 (3.1)	0.778
Malignancy	10 (5.2)	8 (4.2)	0.629
Charlson comorbidity index	3.8 ± 2.6	3.7 ± 2.8	0.747
Clinical parameters at presentation, n (%)			
Total bilirubin > 5 mg/dl	70 (36.6)	73 (38.2)	0.751
Cardiovascular dysfunction	23 (12.0)	19 (9.9)	0.513
Neurological dysfunction	13 (6.8)	11 (5.8)	0.673
Respiratory dysfunction	20 (10.5)	17 (8.9)	0.604
Renal dysfunction	19 (9.9)	13 (6.8)	0.268
Hepatic dysfunction	8 (4.2)	12 (6.3)	0.358
Hematologic dysfunction	8 (4.2)	7 (3.7)	0.792
Cholangitis severity, n (%)			
Severe	44 (23.0)	34 (17.8)	0.204
Moderate	61 (31.9)	86 (45.0)	0.009
Mild	86 (45.0)	71 (37.2)	0.119
Time to ERCP (h), median (IQR)	20 (5, 23)	72 (48, 120)	
Antibiotics, n (%)			
Cephalosporin based	145 (75.9%)	133 (69.6%)	0.168
Piperacillin/tazobactam	10 (5.2%)	11 (5.8%)	0.822
Carbapenem	33 (17.3%)	43 (22.5%)	0.200
Others	3 (1.6%)	4 (2.1%)	0.703

*h* hours, *IQR* interquartile rage

Data are presented as number of patients (%) unless otherwise stated. Mean values are presented as mean ± SD. Median values are presented as median (interquartile range)

### Clinical outcomes in matched cohorts

Among the 382 patients included in the matched analysis, the overall in-hospital mortality rate was 2.4% (*n* = 11), with one death in the urgent ERCP group and ten in the non-urgent ERCP group. Causes of deaths included refractory septic shock secondary to cholangitis (*n* = 8) and respiratory failure due to ventilator-associated pneumonia (*n* = 3).

Urgent ERCP was significantly associated with reduced in-hospital mortality compared to non-urgent ERCP, with an adjusted odds ratio (OR) of 0.09 (95% CI: 0.01–0.73; *p* = 0.024). Rate of persistent organ failure at 72 h was similar between groups (11.5% vs 12.0%, adjusted OR 0.84; 95% CI: 0.44–1.59; *p* = 0.587). Stone clearance rate did not differ significantly (74.3% vs 75.4%, adjusted OR 0.98; 95% CI: 0.61–1.56; *p* = 0.929, nor did rates of procedure-related complications (4.7% vs 4.2%, adjusted OR 1.09; 95% CI: 0.41–2.89; *p* = 0.869). Notably, patients in the urgent ERCP had a shorter median hospital stay compared to those in the non-urgent ERCP group (5 vs 8 days, adjusted OR 0.18; 95% CI: 0.14–0.23; *p* < 0.001). Readmission rates were also comparable between groups, with both reporting 8.1% (adjusted OR 0.98, 95% CI: 0.57–1.59; *p* = 0.952) (Table 3).

**Table 3:** Comparison of clinical outcomes between two groups in matched populations

Outcome	Urgent ERCP	ERCP > 24 h	Unadjusted OR (95% CI)	*p* value	Adjusted OR (95% CI)*	*p* value
	(N = 191)	(N = 191)				
In-hospital mortality	1 (0.5)	9 (2.1)	0.11 (0.01 – 0.85)	0.034	0.09 (0.01 – 0.73)	0.024
Persistent organ failure at 72 h	22 (11.5)	23 (12.0)	0.95 (0.51 – 1.77)	0.874	0.84 (0.44 – 1.59)	0.587
Length of stay (days), median	5 (3.3, 8)	8 (6, 12)	0.19 (0.13 – 0.24)	< 0.001	0.18 (0.14 – 0.23)	< 0.001
Complete stones removal	142 (74.3)	144 (75.4)	0.95 (0.59 – 1.50)	0.814	0.98 (0.61 – 1.56)	0.929
Intervention-related complications	9 (4.7)	8 (4.2)	1.13 (0.43 – 2.99)	0.804	1.09 (0.41 – 2.89)	0.869
Readmission	31 (8.1)	31 (8.1)	1.00 (0.58 – 1.72)	0.998	0.98 (0.57 – 1.59)	0.952

Data are presented as number of patients (%) unless otherwise stated. Median values are presented as median (interquartile range)

*Adjusted for variables with standard difference > 0.15 among the matched population, including age, sex, comorbidities, Charlson comorbidity index, and cholangitis severity according to the Tokyo Guidelines 2018

### Subgroup analysis by disease severity

A subgroup analysis was performed according to disease severity grading based on the TG13/18 classification. In patients with moderate cholangitis, urgent ERCP was significantly associated with reduced in-hospital mortality compared to non-urgent ERCP (0 vs 4 deaths; *p* < 0.01). A similar mortality benefit was observed in the severe subgroup (1 vs 6 deaths; *p* = 0.024). In contrast, no deaths occurred in patients with mild cholangitis in either group. The incidence of persistent organ failure did not different significantly between urgent and non-urgent ERCP groups across all severity categories (Table 4).

**Table 4:** Subgroup analysis according to severity

Outcome		Urgent ERCP, N	ERCP > 24 h, N	*p* value
Severe (*n* = 98)	In-hospital mortality	1	6	0.024
	Persistent organ failure	23	23	0.340
Moderate (*n* = 184)	In-hospital mortality	0	4	< 0.01
	Persistent organ failure	1	5	0.370
Mild (*n* = 173)	In-hospital mortality	0	0	
	Persistent organ failure	0	0	

## Discussion

Acute cholangitis results from bacterial infection superimposed on biliary obstruction [5, 8]. While international guidelines recommend timely biliary drainage according to disease severity, they provide limited guidance on specific timing [7]. Several studies have investigated various thresholds—within 24, 48, or 72 h—with consistent findings that delays beyond 48–72 h are linked to worse outcomes, including increased mortality, persistent organ failure, and longer hospital stays [9–12]. National registry data from 77,323 patients have shown higher in-hospital mortality and longer hospital stays with delayed ERCP, especially after 48 h [13]. Similarly, delayed intervention has been associated with greater healthcare costs, ICU utilization, and 30-day readmission rates [14]. However, the benefits of performing ERCP within 24 h remain less well established.

Some large multicenter studies and meta-analyses suggest a potential mortality benefit in grade II cholangitis with ERCP within 24 h, though not consistently observed in patients with more severe illness [1]. A cohort study of 260 cholangitis patients with septic shock demonstrated reduced mortality when ERCP was performed within 12 h [4]. Other smaller retrospective studies reported trends favoring early intervention but lacked statistical power to confirm mortality benefit [6, 15]. A recent meta-analysis suggested reduced odds of persistent organ failure with ERCP within 24 h, though statistical significance was not achieved [14].

In this study with propensity score-matched cohort, ERCP performed within 24 h of admission was associated with a significant reduction in in-hospital mortality, particularly in patients with moderate to severe cholangitis secondary to choledocholithiasis. The mortality benefit appeared to stem primarily from a reduction in deaths related to septic shock and respiratory failure. Although ERCP within 24 h shortened the duration of hospitalization, it did not significantly influence rates of persistent organ failure, stone clearance, readmission, or ERCP-related complications. Subgroup analysis further confirmed that the survival advantage of ERCP within 24 h was confined to moderate and severe disease, with no differences observed among patients with mild cholangitis.

Our findings align with emerging evidence [2] that ERCP within 24 h confers a survival benefit, especially in patients with moderate to severe disease. In our cohort, the median time to ERCP in the urgent group was 19.5 h from admission, with an interquartile range of 5–23 h. This indicated that while some patients underwent very early intervention within the first several hours, the majority received ERCP later within the 24-h window. These findings underscore the importance of achieving biliary decompression within the first hospital day. The incidence of persistent organ failure in our cohort was lower than previous studies of 15–22% [9, 10] which may have limited power to detect differences. The mortality benefit despite similar organ failure rates may reflect adequate systemic resuscitation and earlier infection control with efficient biliary decompression. The consistent reduction in mortality, along with a shorter hospital stay in the ERCP within 24 h group (median 5 vs 8 days), suggests meaningful clinical and resource-related advantages.

Approximately 75% of patients in this study achieved complete stone clearance despite the urgency of intervention, which aligns with international quality benchmarks [16]. Major ERCP-related complications, including post-ERCP pancreatitis, bleeding, and perforation, were infrequent, reflecting a high standard of procedural safety even in the urgent setting [16, 17].

This study has a few strengths. It focuses exclusively on patients with choledocholithiasis-induced cholangitis, eliminating confounding from malignant or other non-stone etiologies. Propensity score matching was used to balance key clinical variables and reduce confounding, thereby improving the validity of outcome comparisons. However, the retrospective design introduces potential bias from unmeasured confounding, as patients with higher disease burden may have been prioritized for urgent ERCP, potentially biasing results against the early group. The fact that the early group nonetheless demonstrated significantly better survival suggests that early intervention may confer a genuine benefit; however, this possibility of residual confounding should be acknowledged. Another potential confounder relates to antibiotic therapy. Regimens were similar between urgent and non-urgent groups. Although broader agents such as carbapenems were more frequently used in severe cases, distribution within each severity group was balanced, suggesting that antibiotic selection did not account for the observed mortality difference, though synergy with early source control remains possible. Additionally, the relatively small number of mortality events overall, and within certain subgroups—especially mild disease—limits the statistical power for subgroup mortality analyses. As such, conclusions regarding subgroup-specific survival benefit should be interpreted with caution and considered hypothesis-generating rather than definitive. Finally, the focus on choledocholithiasis limits generalizability to patients with other causes of biliary obstruction, such as malignancy, where the disease course and response to intervention differ substantially [18].

In conclusions, this study highlights the clinical benefit of performing ERCP within 24 h in patients with moderate to severe acute cholangitis due to choledocholithiasis. Thus, the ERCP service for this patient population should focus on severity-based triage protocols and reliable access to endoscopy with biliary drainage within the first day of presentation, particularly in resource-limited settings. Further prospective studies are warranted to validate these findings and to refine the optimal timing of ERCP across different clinical contexts and etiologies.

## Supplementary Information

Below is the link to the electronic supplementary material.

Supplementary file1 (DOCX 30 KB)
